# Preparation of a new fixed-combination carteolol-netarsudil ophthalmic solution via one-pot pH-adjusting solubilization strategy for glaucoma treatment

**DOI:** 10.1080/10717544.2026.2708376

**Published:** 2026-08-15

**Authors:** Dian Jiao, Tao Wang, Tu Lu, Xiaohua Ran, Yanxin Li, Yanhua Liu, Chunyang Zhao, Tianhong Zhang, He-Nan Liu, Jin Sun, Xiaoli Ma, Qikun Jiang

**Affiliations:** a Department of Pharmaceutics, Wuya College of Innovation, Shenyang Pharmaceutical University, Shenyang, China; b Department of Ophthalmology, The First Hospital of China Medical University, Shenyang, China; c Department of Pharmaceutics, School of Pharmacy, Ningxia Medical University, Yinchuan, China; d Department of Ophthalmology, Shengjing Hospital of China Medical University, Shenyang, China; e Department of Pharmacy, The First Hospital of China Medical University, Shenyang, China; f Joint International Research Laboratory of Intelligent Drug Delivery Systems, Ministry of Education, Shenyang, China

**Keywords:** Fixed-combination ophthalmic solution, carteolol, netarsudil dimesylate, thickener, one-pot pH-adjusting solubilization strategy, glaucoma

## Abstract

Currently, the commercial fixed-combination of two agents is facing several clinical challenges, including suboptimal intraocular pressure (IOP) reduction, which requires multiple daily administrations, compromised bioavailability due to rapid tear film clearance and nasolacrimal drainage, and preservative-induced ocular surface toxicity. To address these limitations, we intend to develop a novel preservative-free, long-acting fixed-combination formulation of carteolol hydrochloride and netarsudil dimesylate. This once-daily formulation demonstrates enhanced therapeutic potential through optimized physicochemical stability and extended ocular residence time in theory. However, commercialized netarsudil dimesylate is precipitated when the pH of the solution is above 5.4 or when carteolol hydrochloride is mixed with netarsudil dimesylate. Consequently, the preservative-free and long-acting fixed-combination carteolol-netarsudil containing hydroxypropyl methylcellulose (HPMC) as a thickener (FC-CN-TKN) was prepared in a pH about 5.0 solution using an extremely simple one-pot pH-adjusting solubilization strategy, achieving stable co-solubilization of both active pharmaceutical ingredients. The optimized FC-CN-TKN formulation demonstrated excellent physicochemical stability under refrigerated storage conditions. Additionally, FC-CN exerted no influence on the intraocular penetration of each active compound in the pharmacokinetic study, and HPMC-mediated viscosity enhancement significantly prolonged precorneal retention, further improving its residence time and bioavailability. Importantly, once daily, FC-CN-TKN exerted a potent IOP-lowering effect and protective effect on retinal ganglion cells. The innovative FC-CN-TKN was stable, safe, and effective, being a promising glaucoma therapy.

## Graphical abstract

**Figure:**
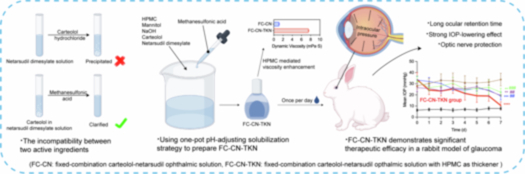

Schematic illustration of a one-pot pH-adjusting solubilization strategy with methanesulfonic acid enables fixed-combination carteolol-netarsudil ophthalmic solution with a thickener (FC-CN-TKN): overcoming drug–drug incompatibility for enhanced ocular retention time, intraocular pressure (IOP) reduction, and neuroprotection.

## 1. Introduction

Glaucoma, a progressive neurodegenerative disorder with a multifactorial etiology, ranks as the second leading cause of global blindness (Kupfer 1994; Arthur and Cantor 2011). Elevated intraocular pressure (IOP) represents the primary modifiable risk factor, exerting mechanical stress on retinal neurons. This pathological compression initiates a cascade of degenerative events, including retinal ganglion cell apoptosis, thinning of the nerve fiber layer, and eventual irreversible vision loss (Vrabec and Levin 2007; Goel et al. 2010; Babić 2015; Marshall et al. 2018; Garcia-Medina et al. 2020; Russo et al. 2022). The mechanisms of lowering IOP include reducing aqueous humor (AH) production and increasing AH outflow. Strategies for the clinical management of glaucoma include drug treatment and non-drug treatment (laser treatment and surgical treatment) (Storgaard et al. 2021). The high risk, serious adverse reactions, and high medical costs of non-drug therapy limit its clinical application. Owing to its high safety and convenience, pharmacotherapy has become the most widely used method in the clinic (Guglielmi et al. 2019; Webb 2021). According to the different IOP-lowering mechanism, modern antiglaucoma agents are classified into the following principal categories: β-adrenergic blockers, prostaglandin analogs, carbonic anhydrase inhibitors, adrenergic agonists, and Rho-associated kinase (ROCK) inhibitors (Ostler et al. 2021; Patton and Lee 2024). While monotherapy demonstrates efficacy in early-stage disease, advanced glaucoma frequently necessitates multiple administrations due to the ceiling effect of single-agent IOP reduction—a scenario exacerbating systemic exposure to medications and compromising adherence through complex dosing regimens.

Currently, the clinically used fixed-combination two agents can be divided into two classes: prostaglandin-containing formulations and prostaglandin-free alternatives. While prostaglandin analogs effectively lower intraocular pressure (IOP), their therapeutic profile is marred by adverse effects, including iris pigmentation and periocular hypertrichosis (Johnstone 1997; Watson 1998). Importantly, the inherent hydrophobicity of prostaglandins necessitates solubilizing agents (e.g. polysorbate 80 and hydrogenated castor oil) and preservatives (e.g. benzalkonium chloride) in aqueous formulations, which significantly increase ocular side effects such as keratitis, conjunctival allergy, and subconjunctival tissue fibrosis, resulting in significantly lower patient compliance (Kaur et al. 2009; Rosin and Bell 2013; Thygesen 2018). These limitations underscore the clinical imperative for prostaglandin-free combination therapies. Currently, many preparations without prostaglandins are marketed, such as Cospt® (2% dorzolamide hydrochloride-0.5% timolol maleate), Combigan® (0.2% brimonidine tartrate-0.5% timolol maleate), and Simbrinza® (1% brinzolamide-0.5% brimonidine tartrate), etc. Repeated administration, preservative exposure, and poor efficacy have limited their clinical application. Thus, a fixed combination of a β-adrenergic blocker and a ROCK inhibitor with multiple IOP-lowering mechanisms involving decreasing AH production and increasing AH outflow may potentially be effective in synergistically lowering IOP, protecting the optic nerve, and delaying blindness.

**Figure 1.**
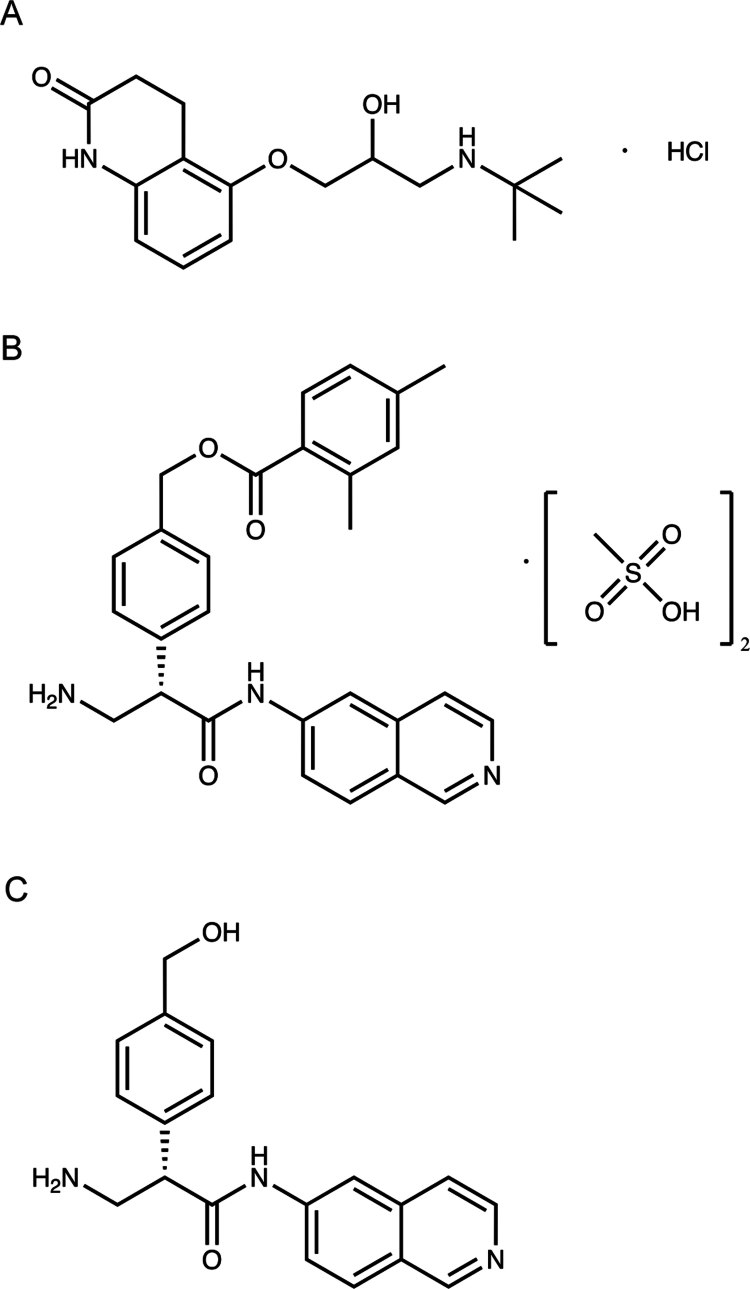
Chemical structures of carteolol hydrochloride (A), netarsudil dimesylate (B), and netarsudil-M1 (C) used in this study.

Carteolol hydrochloride (Figure 1A), a non-selective β-adrenergic blocker, exerts potent IOP-lowering effects through the suppression of aqueous humor (AH) production (Gorgone et al. 1983; Sugiyama et al. 1989). Complementarily, netarsudil dimesylate (Figure 1B) can be absorbed by the cornea and metabolized to the active metabolite netarsudil-M1 (Figure 1C), which increases the outflow of AH to exert an IOP-lowering effect via Rho kinase inhibition (Rao and Epstein 2007; Dasso et al. 2018). Importantly, netarsudil has an optic nerve-protecting effect to delay blindness. Thus, a potentially effective carteolol (2%)-netarsudil (0.02%) ophthalmic solution (FC-CN) was designed to reduce IOP through a dual mechanism and protect neural nerves (Rao and Epstein 2007; Dasso et al. 2018; Lin et al. 2018). It was found that FC-CN showed poor stability under the following two conditions: (1) pH is an important factor to keep the ophthalmic solution stable. Commercially available netarsudil dimesylate ophthalmic solution (Rhopressa®) was provided as a sterile solution with a pH of about 5.0. Indeed, the commercialized netarsudil dimesylate was precipitated when the pH of the solution was above 5.4 (Wang et al. 2022). (2) Carteolol hydrochloride, the salt of commercial carteolol, was not compatible with commercial netarsudil dimesylate, causing white precipitation at a pH about 5.0 solution. Notably, no prior studies have resolved these incompatibility issues while maintaining iso-osmolality and ocular tolerability to prepare FC-CN. Furthermore, FC-CN has a short ocular retention time due to tear dilution. Thus, it still faces significant challenges to build a stable and long-acting fixed-combination ophthalmic solution.

Building upon these situations, we developed a novel fixed-combination carteolol and netarsudil dimesylate ophthalmic solution with hydroxypropyl methylcellulose (HPMC) as a thickener (FC-CN-TKN) to prolong the retention time of drugs in the eye, thereby increasing the penetration and absorption effect of drugs, aiming to gain a better IOP-lowering effect and vision protection. In order to prevent the carteolol hydrochloride from causing the precipitation of netarsudil dimesylate, free carteolol was selected, and a one-pot pH-adjusted solubilization strategy was used, that is, methanesulfonic acid was used as a pH regulator to adjust the pH of the solution to about 5.0 to achieve the co-solubilization of carteolol and netarsudil dimesylate and solve the problem of incompatibility between carteolol hydrochloride and netarsudil dimesylate. FC-CN-TKN showed good stability during refrigeration storage. Additionally, FC-CN-TKN exerted no influence on the intraocular penetration of each active compound in the pharmacokinetic study, and the use of HPMC could effectively increase the viscosity of FC-CN-TKN, further improving its residence time and bioavailability. Importantly, once daily, FC-CN-TKN exerted a potent IOP-lowering effect and protective effect on retinal ganglion cells. The FC-CN-TKN was stable, safe, and effective, being a promising glaucoma therapy.

## 2. Materials and methods

### 2.1. Materials and instruments

Carteolol was purchased from Shanghai Rechemscience Co., Ltd. Carteolol hydrochloride was purchased from the China National Institutes for Food and Drug Control. Netarsudil dimesylate was purchased from Shanghai Pharmaceutical Co., Ltd. Mannitol, carbomer and sodium alginate was purchased from Shanghai Macklin Biochemical Technology Co., Ltd. Methanesulfonic acid was purchased from Tianjin Hengxing Chemical Reagents Co., Ltd. Different types of hydroxypropyl methylcellulose (HPMC) and carboxymethylcellulose sodium (CMC-Na) were purchased from Anhui Sunhere Pharmaceutical Excipients Co., Ltd. Polyvinyl pyrrolidone (PVP) was bought from Tianjin Bodi Chemical Co., Ltd. Sodium Hyaluronate (HA) was bought from Bloomage Biotech Co., Ltd. Artificial tears HPMC ophthalmic solution (Aierming®) was bought from Shenyang Xingqi Pharmaceutical Co., Ltd. Artificial tears HPMC ophthalmic solution (Zhendi®) ophthalmic solution was bought from Changchun Dirui Pharmaceutical Co., Ltd. Carteolol hydrochloride ophthalmic solution (Mikelan®) was bought from China Otsuka Pharmaceutical Co., Ltd. PHS-3C pH meter was bought from Tianjin Taisite Instrument Co., Ltd. SHH-500SD-2T drug stability test chamber was bought from Chongqing Yongsheng Instrument Co., Ltd. AR2000ex rheometer was bought from TA Instrument Co., Ltd. Icare TA01I tanometer was bought from Qisheng (Shanghai) medical device Co., Ltd. All other reagents and chemicals used in this study were of analytical grade.

### 2.2. Selection of pH

Maintaining physiochemical stability of active pharmaceutical ingredients (APIs) requires precise pH control—a critical determinant for ophthalmic formulation efficacy and tolerability. To systematically assess the pH-dependent stability profiles, carteolol, carteolol hydrochloride, or netarsudil dimesylate were dissolved in different pH of deionized water or FC-CN with or without methanesulfonic acid. HPLC was used to determine the content of carteolol and netarsudil in order to evaluate the stability of carteolol and netarsudil at different pH values.

### 2.3. One-pot pH-adjusting solubilization strategy

Drug incompatibility compromises the safety and effectiveness of the combined drugs. We found that carteolol hydrochloride would cause netarsudil dimesylate to precipitate at approximately pH 5.0 for better clarity. Therefore, carteolol (free base) was used in this study to avoid the incompatibility of carteolol hydrochloride and netarsudil dimesylate. However, carteolol is insoluble in water. Here, a simple preparation process called a one-pot pH-adjusting solubilization strategy was proposed to solve the incompatibility between carteolol hydrochloride and netarsudil dimesylate. Methanesulfonic acid, a commercial salt of netarsudil, was chosen as a pH regulator and to solubilize carteolol. The content of carteolol in solution was evaluated using HPLC.

### 2.4. Selection of thickener

Suitable thickeners can prolong the retention time of drugs on the ocular surface and increase the drug permeation, thereby improving the treatment effect. Several common thickeners for ophthalmic solutions were screened at the amount of 0.1 w/v%. Carbomer, HPMC, CMC-Na, HA, sodium alginate, and PVP were dissolved in FC-CN to observe whether white precipitation appeared to investigate the compatibility between thickeners and active ingredients. Different types of HPMC were screened at the amount of 0.5 w/v% to evaluate whether the viscosity of FC-CN was similar to that of commercial artificial tear HPMC ophthalmic solutions (Aierming® and Zhendi®). The viscosity of the solutions was measured with a rheometer.

### 2.5. Preparation of ophthalmic solutions

#### 2.5.1. Preparation of netarsudil dimesylate ophthalmic solution

On account of the unavailability of commercial netarsudil dimesylate ophthalmic solution in China, self-made preparations are used because of their relatively simple prescription composition and preparation process (Lin et al. 2018). The preparation process is as follows: boric acid (0.05 g), mannitol (1.2 g), and netarsudil dimesylate (0.0285 g) were dissolved in 95 mL of deionized water. The pH value was adjusted to about 5.0 with 1 mol·L^−1^ sodium hydroxide solution, and deionized water was added to 100mL to obtain a netarsudil dimesylate ophthalmic solution. The solution was encapsulated in ampoules.

#### 2.5.2. Preparation of carteolol ophthalmic solution

Boric acid (0.05 g), mannitol (1.2 g), and carteolol (2.0 g) were dissolved in 90  mL of deionized water. A total of 710  μL of methanesulfonic acid was added, and stirred for 20 minutes. Then HPMC (SH-E50, 0.5 g) was added and stirred until it was completely dissolved. The pH value was adjusted to about 5.0 with a 1 mol·L^−1^ sodium hydroxide solution, and deionized water was added to 100 mL to obtain a carteolol ophthalmic solution. The solution was encapsulated in ampoules.

#### 2.5.3. Preparation of FC-CN-TKN

Boric acid (0.05 g), mannitol (1.2 g), and carteolol (2.0 g) were dissolved in 90  mL of deionized water. A total of 710 μL of methanesulfonic acid was added, and stirred for 20 minutes. 0.0285 g of netarsudil dimesylate was added, and stirred for 1 minute. Then HPMC (SH-E50, 0.5 g) was added and stirred until all the compounds were completely dissolved. The pH value was adjusted to about 5.0 with a 1 mol·L^−1^ sodium hydroxide solution, and deionized water was added to 100 mL to obtain FC-CN-TKN. The solution was encapsulated in ampoules.

### 2.6. *In vitro* stability studies

The regulatory expectations for ophthalmic products have always been the most demanding; meanwhile, the stability of ophthalmic drugs is an important parameter related to the storage condition (Matthews and Wall 2000). According to commercial netarsudil dimesylate ophthalmic solution and commercial carteolol hydrochloride ophthalmic solution (Mikelan®), three different conditions of transportation temperature (40 °C ± 2 °C), room temperature (25 °C ± 2 °C), and refrigeration temperature (5 °C ± 3 °C) were selected to evaluate the *in vitro* stability of FC-CN-TKN. The content of the active ingredients, pH value, dynamic viscosity, and osmolality after being stored under the different conditions described above for a period of time were measured to evaluate the stability of FC-CN-TKN.

### 2.7. Animal study

All the animal studies were performed in accordance with the ARRIVE guidelines and were conducted according to the Guidelines for the Care and Use of Laboratory Animals and were approved by the Institutional Animal Care and Use Committee (IACUC) of Shenyang Pharmaceutical University. Healthy male Japanese white rabbits [SYXK (Liao) 2018-0009] (weighing 1.8–2.2 kg) were purchased from Liaoning Changsheng Biotechnology Co., Ltd. (Benxi, China). They were kept in individual cages at a temperature of 22 °C ± 2 °C and a relative humidity of 40%–70%. They were fed with balanced diet pellets and had free access to food and water. The eyes of all rabbits were examined prior to the experiment, and only the animals without any ocular disease were used for the experiments.

#### 2.7.1. *In vivo* pharmacokinetic study

Seventy-two healthy male Japanese white rabbits (1.8–2.2 kg) were kept in individual cages at a temperature of 22 °C ± 2 °C and a relative humidity of 40%–70% for 7 days. They were fed with balanced diet pellets and had free access to food and water. After seven days, they were randomly divided into 4 groups of 18 rabbits in each group. Each group was divided into 9 subgroups (2 rabbits for each time point). Each rabbit was treated with 40 μL of FC-CN-TKN, FC-CN, carteolol hydrochloride ophthalmic solution, or netarsudil dimesylate ophthalmic solution in the lower conjunctival cul-de-sac of both eyes. The eyelids were kept closed for 10 s after administration to prevent the loss of the instilled drug solution. After administration for 0.083 hours, 0.25 hours, 0.5 hours, 1 hour, 1.5 hours, 2 hours, 4 hours, 8 hours, and 24 hours, the rabbits were euthanized by exsanguination under deep anesthesia. After that, the eyeballs were removed and dissected into the cornea and AH. The cornea was separately weighed at the time of collection, and all the samples (cornea and AH) were separately stored in separate tubes, frozen immediately, and stored at −80 °C until the analysis began.

Netarsudil is an ester prodrug that is quickly hydrolyzed to netarsudil-M1 by corneal esterases after ocular administration (Sjöquist and Stjernschantz 2002; Lin et al., 2018). Therefore, the contents of carteolol, netarsudil, and netarsudil-M1 were evaluated in all the collected samples by ultraperformance liquid chromatography‒tandem mass spectroscopy (UPLC-MS/MS). The pharmacokinetic parameters, including the time to maximum concentration (T_max_), maximum observed concentration (C_max_), and area under the concentration time curve from zero to the time of last non-zero concentration (AUC_0−t_), were calculated with DAS 2.0.

#### 2.7.2. *In vivo* pharmacodynamic study

To verify whether the addition of HPMC improved the therapeutic effect, the IOP-lowering efficacy of FC-CN-TKN was evaluated in a high IOP rabbit model and compared with two monotherapy agents, carteolol hydrochloride ophthalmic solution and netarsudil dimesylate ophthalmic solution, and FC-CN. Thirty Japanese white rabbits (1.8–2.2 kg) were acclimatized for 7 days before the start of the experiments. Then, all rabbits were first anesthetized with an intravenous injection of 20 w/v% urethane solution (4 ml/kg, converted to 0.8 g/kg urethane eq.) (Hara and Harris 2002), 100 μL AH was removed from the anterior chamber of the right eye using a disposable syringe, and then slowly injected 100 μL of the compound carbomer solution (0.3 w/v %) through a disposable syringe (Ou-Yang et al. 2020). After the procedure, all the eyes were treated with a drop of levofloxacin hydrochloride ophthalmic solution (0.3%). A tonometer (Icare® TA01I, Finland) was used to measure the IOP value once a day and 6 times per eye. The tonometer slowly approached the rabbit eye until the probe was about 4 mm away from the cornea. Press measurement button to measure and the average values were recorded. When the IOP of all the right eyes was higher than 22 mmHg for 3 d consecutively, once-daily carteolol hydrochloride ophthalmic solution, netarsudil dimesylate ophthalmic solutions, FC-CN or FC-CN-TKN was administered into the lower conjunctival sac of the eyes in each treatment group for 7 days. Rabbits did not receive any treatment in the right eyes of the model group. And all the left eyes without any treatment were used as a control. Continuously measure the IOP of all rabbits once a day and 6 times per eye. Graphs of the changes in average IOP values and IOP changes during the administration period were drawn to describe the IOP-lowering effect of FC-CN-TKN.

#### 2.7.3. Histological examination

Extensive studies have reported that ROCK inhibitors increased ocular blood flow, which may slow down the progression of glaucomatous optic neuropathy by directly increasing the perfusion of the retina and optic disk (Tanna and Johnson 2018; Moura-Coelho et al. 2019). The rabbits were euthanized by exsanguination under deep anesthesia after the pharmacodynamic study, and the eyeballs were removed and fixed in 4% paraformaldehyde to evaluate the potential effect of FC-CN-TKN on rabbit's optic nerve. The obtained tissue sections of the retina were sliced using a microtome and stained with hematoxylin and eosin (H&E) and TUNEL technique. The retinas from each animal were imaged, and Imaging-Pro-Plus 6.0 software (Media Cybernetics Inc., Silver Spring, MD) was used to quantify the number of retinal ganglion cells (RGCs).

### 2.8. Statistical analysis

All the statistical analyses were carried out using SPSS software version 24. All the experiments were performed in triplicate, and the results were presented as the mean ± standard deviation (SD). The Welch's *t*-test, Mann–Whitney *U*-test, or one-way analysis of variance (ANOVA) were used to analyze the data in the case of two groups or multiple groups, respectively. A value of *p* < 0.05 was considered statistically significant.

## 3. Results and discussion

### 3.1. Incompatibility between carteolol hydrochloride and netarsudil dimesylate and one-pot pH-adjusting solubilization strategy

**Figure 2.**
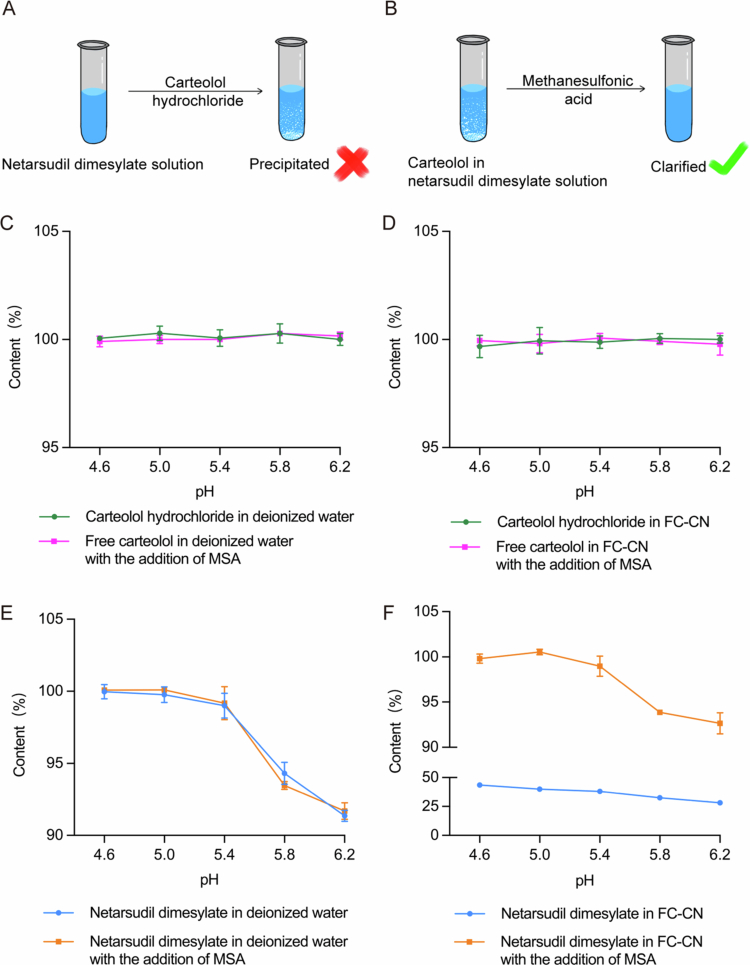
One-pot pH-adjusting solubilization strategy with methanesulfonic acid (MSA) solved the incompatibility between carteolol hydrochloride and netarsudil dimesylate. (A) Carteolol hydrochloride was incompatible with netarsudil dimesylate. (B) Free carteolol has a poor solubility. The use of MSA can increase the solubility of carteolol in aqueous solution. (C, D) Content of carteolol or carteolol hydrochloride in deionized water or FC-CN as the pH changed. (E, F) The content of netarsudil dimesylate in deionized water or FC-CN as the pH changed. Data are presented as the mean ± SD (*n* = 3).

The therapeutic efficacy and safety profile of fixed-combination ophthalmic formulations are critically dependent on both active ingredient compatibility and precise pH regulation. In the present study, we first found that carteolol hydrochloride, the salt of commercial carteolol, was not compatible with commercialized netarsudil dimesylate in solution. White precipitation appeared and the content of netarsudil dimesylate was below 50% in pH 4.6–6.2 solution (Figure 2A, F). Further analysis identified pH sensitivity as a critical destabilizing factor, with netarsudil solubility decreasing as the pH increases, while carteolol hydrochloride was not affected (Figure 2C, E). Therefore, free carteolol may be a better choice for preparing FC-CN, and the pH value of FC-CN-TKN should be controlled to be approximately 5.0 to maintain the stability of the solution.

The formulation development encountered a critical solubility challenge: the free base form of carteolol exhibited negligible solubility (<10% dissolution efficiency) in pH 5.0 aqueous solutions (Figure S1A), rendering conventional preparation methods ineffective. To overcome this limitation, a one-pot pH-adjusting solubilization strategy was proposed to solve the problem of catechol insolubility. Methanesulfonic acid (MSA) was chosen as the pH regulator to adjust the solution pH to about 5.0 to increase the solubility of free carteolol. As shown in Figure 2B, C, D, and S1B, when the pH of the solution was around 5.0, the solution became clear, and the content of carteolol was above 98%. Furthermore, MSA will not affect the stability of netarsudil dimesylate in both deionized water and FC-CN (Figure 2E, F). This strategy solves the problem of incompatibility between carteolol hydrochloride and netarsudil dimesylate, establishing a robust platform for developing FC-CN-TKN.

### 3.2. Selection and dosage of thickener

**Figure 3.**
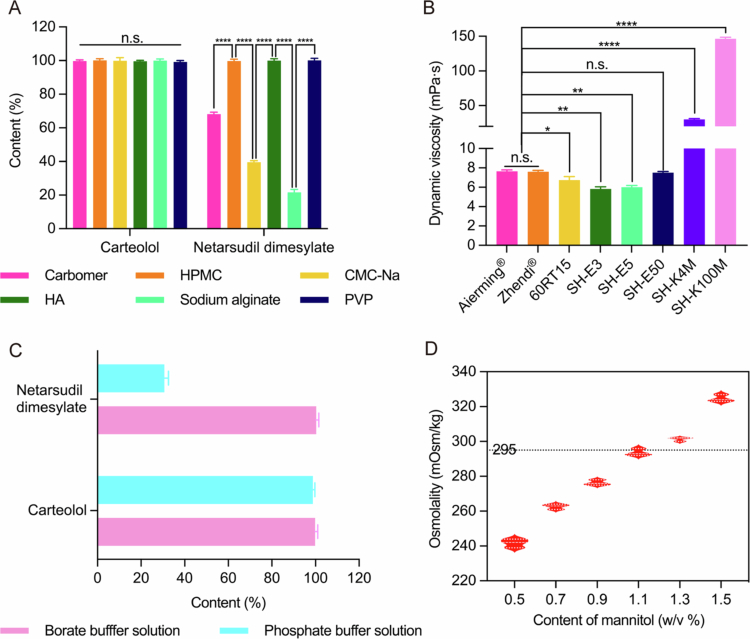
The appropriate selection of excipients and the scientific development of the prescription critically determine the ultimate quality of FC-CN-TKN. (A) Content of carteolol and netarsudil in FC-CN with different thickeners. (B) The dynamic viscosity of FC-CN with different types of HPMC. (C) Content of carteolol and netarsudil dimesylate in borate buffer solution and phosphate buffer solution. (D) The osmolality of the solution when mannitol was used as a tonicity agent at different concentrations. All data are presented as the mean ± SD (*n* = 3). *p* value: n.s.: no significance, *p < 0.05, **p < 0.01, ***p < 0.001, and ****p < 0.0001.

The therapeutic efficacy of ophthalmic solutions is inherently limited by rapid precorneal clearance, with >90% of instilled drugs are typically eliminated within 5 minutes through nasolacrimal drainage. Currently, thickeners are widely used in the field of ophthalmic drugs, which prolong the drug retention time and delay the elimination of active ingredient in the eye, thereby reducing the frequency of ophthalmic solution administration and increasing patient tolerance. Thickeners commonly used in the clinic include carbomer, hydroxypropyl methylcellulose (HPMC), sodium hyaluronate (HA), carboxymethylcellulose sodium (CMC-Na), polyvinyl pyrrolidone (PVP), and sodium alginate (Ludwig 2005; Pahuja et al. 2012). First, the formulation screening evaluated six mucoadhesive agents (0.1 w/v%) for compatibility with the FC-CN system. As shown in Figures 3A and S2, the results demonstrated that the carbomer, CMC-Na, and sodium alginate could cause the precipitation of netarsudil dimesylate. Among other ones, HPMC is a substance that can form a viscous solution when dissolved in water, and its properties are similar to those of the viscoelastic substances (mainly mucins) in tears. Therefore, it is often used as artificial tears. Also, HPMC is non-toxic, inexpensive, and easy to obtain. Importantly, the solubility of HPMC in water is not affected by pH. Therefore, HPMC was preliminarily chosen as a thickener in FC-CN-TKN.

Currently, the viscosity of the ophthalmic solution and the dosage of thickener are not specified. The dosage of HPMC for the commercially available artificial tears HPMC ophthalmic solutions was 0.5  w/v%. The viscosity of the preparations with 0.5 w/v % different types of HPMC (60RT15, SH-E3, SH-E5, SH-E50, SH-K4M, and SH-K100M) was determined and also compared with those of artificial tears HPMC ophthalmic solutions. As shown in Figure 3B and Table S1, HPMC (SH-E50) demonstrated ideal viscosity matching (7.52 ± 0.15 mPa·s vs. 7.63 ± 0.12 mPa·s for commercial references). This systematic approach established 0.5 w/v% HPMC (SH-E50) as the thickener of FC-CN-TKN.

### 3.3. Preparation of FC-CN-TKN

First, the selection of appropriate buffering agents is critical for maintaining ophthalmic solution stability while minimizing ocular surface irritation. Refer to the commercially available carteolol hydrochloride ophthalmic solution and netarsudil dimesylate ophthalmic solution, the buffer solution is borate buffer or phosphate buffer solution. However, netarsudil dimesylate precipitated in the phosphate buffer solution, but borate buffer solution would not influence the stability of both carteolol and netarsudil dimesylate (Figure 3C). Consequently, borate buffer was adopted to ensure formulation stability while providing adequate buffer capacity.

Second, the osmolality of FC-CN-TKN should be equal to that of tears to avoid ocular irritation due to differences in tension. Commercial reference products such as carteolol hydrochloride ophthalmic solution and netarsudil dimesylate ophthalmic solution utilize sodium chloride or mannitol as tonicity agents. Preliminary screening revealed that sodium chloride was incompatible with netarsudil dimesylate and caused the precipitation of netarsudil dimesylate. Mannitol demonstrated superior chemical inertness, with an osmolality‒concentration linear correlation enabling precise adjustment. Thus, mannitol was chosen as the tonicity agent in this study. The osmolality of different concentrations of mannitol solution is shown in Figure 3D. These results showed that when the concentration of mannitol was between 1.1 and 1.3 w/v%, the osmolality of FC-CN-TKN was approximately equal to commercial carteolol hydrochloride ophthalmic solution and netarsudil dimesylate ophthalmic solution (Table S2, Figure S3).

Based on the situations above, referring to commercial carteolol hydrochloride ophthalmic solution and netarsudil dimesylate ophthalmic solution, carteolol (2 w/v%) and netarsudil dimesylate (0.0285 w/v%) were chosen as the active ingredients in this study, and the ophthalmic solution was prepared in borate buffer solution using one-pot pH-adjusting strategy. Methanesulfonic acid (0.71%) was used as a pH regulator to adjust the pH value and solubilize carteolol. HPMC (0.5  w/v%) was used as a thickener to prolong the ocular retention time of the ophthalmic solution and improve the treatment effect. Mannitol (1.2 w/v%) was used as a tonicity agent to maintain the isotonic properties of the preparation with tears. The pH value was finally adjusted to approximately 5.0 with sodium hydroxide aqueous solution (1 mol·L^−1^).

### 3.4. *In vitro* stability study of FC-CN-TKN

**Figure 4.**
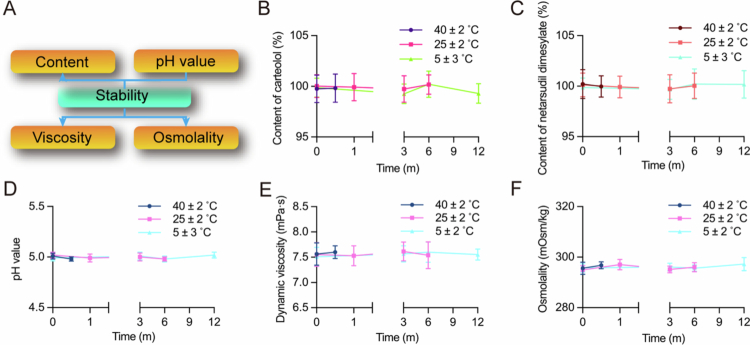
The prepared FC-CN-TKN demonstrated good stability. (A) The stability of FC-CN-TKN was evaluated from various dimensions. The content of carteolol (B), netarsudil dimesylate (C) in FC-CN-TKN, the pH value (D), dynamic viscosity (E), and osmolality (F) of FC-CN-TKN after being stored under different conditions for a period of time. All data are presented as the mean ± SD (*n* = 3).

Storage stability is an important index to evaluate the quality of ophthalmic solution, including refrigeration temperature condition (5 ± 3 °C), room temperature condition (25 °C ± 2 °C), and transportation temperature condition (40 ± 2 °C). According to the prescribing information of those two commercial monotherapy agents (carteolol hydrochloride ophthalmic solution and netarsudil dimesylate ophthalmic solution), both of them can be stored at 2 °C–8 °C for 24 months and can be stored at room temperature for up to 6 weeks after opening. During shipment at temperature up to 40 ℃, both of them should be maintained to avoid exceeding 14 d. The stability profiles of FC-CN-TKN exposed to the above temperature conditions for a period of time are presented in Figure 4. The content of carteolol and netarsudil dimesylate was not significantly different from monotherapy agents (*p* > 0.05). All ophthalmic solutions still contained more than 98% of the initial carteolol and netarsudil dimesylate in refrigeration storage for 12 months (Figure 4A, B). After being stored at 25 ± 2 °C for 6 months, the content of both active ingredients showed no significant changes (Figure 4A, B), fully met the storage requirements after being opened. Besides, FC-CN-TKN was still stable after being stored at 40 ± 2 °C for 14 days (Figure 4A, B), meeting the transportation requirements for ophthalmic solutions. Furthermore, the pH, osmolality, and viscosity value showed no significant difference compared with Day 0 (Figure 4C, D, E), and no visible precipitation formed in all the ophthalmic solutions mentioned above. The demonstrated chemical and physical stability, coupled with preserved therapeutic potency, underscores its viability as a promising antiglaucoma therapy.

### 3.5. *In vivo* pharmacokinetic study of FC-CN-TKN

**Figure 5.**
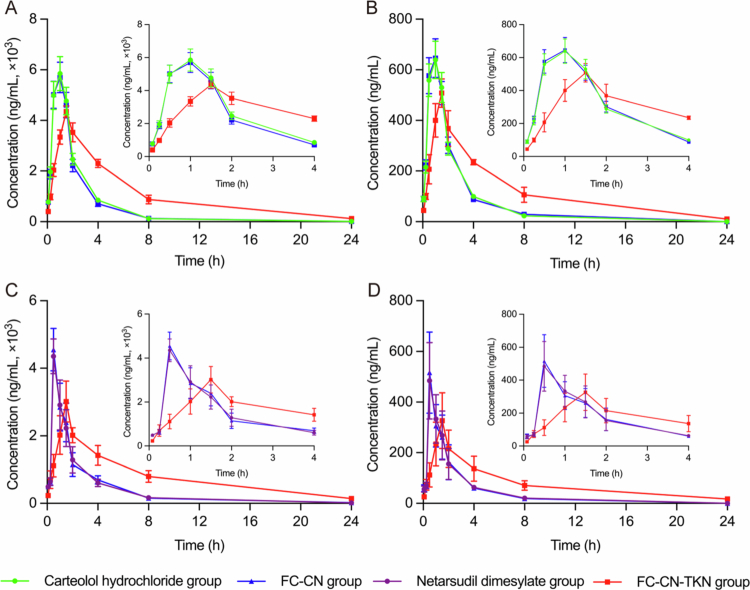
With the addition of HPMC as a thickener, the ocular retention time of FC-CN-TKN increased compared with that of the monotherapy groups and FC-CN group. Cornea and AH concentration‒time curves of carteolol (A, B), netarsudil-M1 (C, D), following a single 40 μL carteolol hydrochloride ophthalmic solution, netarsudil dimesylate ophthalmic solution, FC-CN, or FC-CN-TKN in healthy male Japanese white rabbits. The data are presented as the mean ± SD (*n* = 4).

To evaluate whether the addition of the thickener HPMC would prolong the ocular retention time compared with monotherapy and preparations without a thickener, a comparative pharmacokinetic study was conducted in healthy male Japanese white rabbits to assess the ocular retention enhancement conferred by hydroxypropyl methylcellulose (HPMC) in FC-CN-TKN using monotherapy (carteolol hydrochloride ophthalmic solution and netarsudil dimesylate ophthalmic solution) and FC-CN controls. For each drug, penetration from the cornea into AH is essential to exert the effect of lowering IOP (Isobe et al. 2016). Therefore, it is significant to evaluate the concentration of each drug in both the cornea and AH. Carteolol predominantly remains intact during ocular transit (Gorgone et al. 1983; Sugiyama et al. 1989), while netarsudil undergoes rapid biotransformation to its active metabolite netarsudil-M1 via corneal esterase-mediated hydrolysis (Sjöquist and Stjernschantz 2002; Lin et al. 2018). Thus, the concentration of carteolol, netarsudil, and netarsudil-M1 was measured in both the cornea and AH using UPLC-MS/MS. Notably, netarsudil concentrations fell below quantifiable limits (1  ng/mL) across all timepoints, confirming near-complete presystemic metabolism, which was consistent with previous studies (Wang et al. 2022). Tables S3 and S4 summarize the pharmacokinetic parameters of carteolol and netarsudil-M1 in the cornea and AH. As the thickener was added, the T_max_ delayed (1.0 h vs. 1.5 h for carteolol; 0.5 h vs. 1.5 h for netarsudil-M1) and the AUC _(0−t)_ expanded 1.84 times and 2.01 times compared with those of the carteolol hydrochloride group and the netarsudil dimesylate group (Figure 5B, D), respectively, in AH. This finding indicated that the addition of the thickener HPMC increased the ocular retention time of FC-CN-TKN, increased the amount of drug penetrating through the cornea, and prolonged the duration of drug action, which may provide a better IOP-lowering effect. Additionally, the pharmacokinetic behavior of FC-CN-TKN in the cornea also supported the opinion above (Figure 5A, C).

### 3.6. IOP-lowering efficacy of FC-CN-TKN

**Figure 6.**
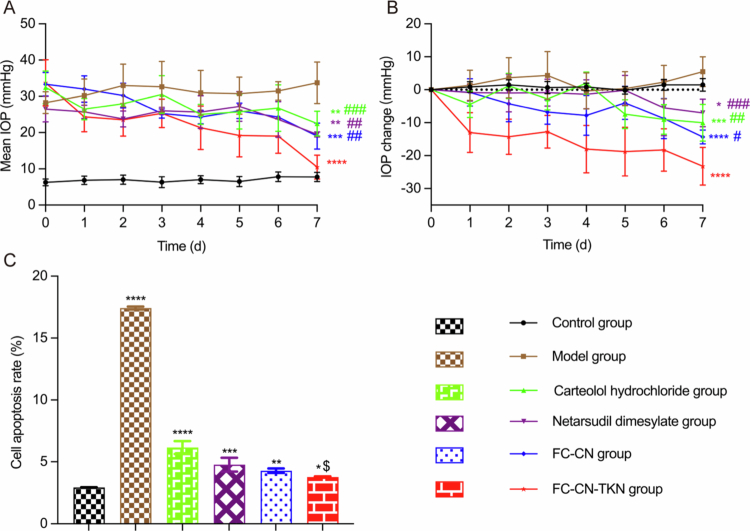
FC-CN-TKN exhibited a good IOP-lowering effect and optic nerve protection ability. The results of the mean actual IOP (A) and IOP change (B) in the control group, model group, carteolol hydrochloride group, netarsudil dimesylate group, FC-CN group and FC-CN-TKN group in rabbits with compound carbomer solution-induced ocular hypertension for 7 d consecutively. (C) Cell apoptosis rate of retinal ganglion cells in the control group, model group, carteolol hydrochloride group, netarsudil dimesylate group, FC-CN group, and FC-CN-TKN group. All data are presented as the mean ± SD (*n* = 3). *p* value: *p < 0.05, **p < 0.01, ***p < 0.001, ****p < 0.0001 compared with the model group, ^#^ *p* < 0.05, ^##^ *p* < 0.01, ^###^ *p* < 0.001 compared with FC-CN-TKN group, ^$^ *p* < 0.05 compared with FC-CN group.

A high IOP is not the sole factor causing damage to the glaucomatous optic nerve, it represents a modifiable risk factor and a quantitative means of measuring the treatment effect (Goel et al. 2010). To assess the IOP-lowering potential of FC-CN-TKN, we conducted comparative pharmacodynamic studies in a carbomer-induced ocular hypertension rabbit model, benchmarking against commercial monotherapies (carteolol hydrochloride ophthalmic solution and netarsudil dimesylate ophthalmic solution) and FC-CN. The mean actual IOP and mean IOP change from the baseline in each group are shown in Figure 6A, B, and Table S5. These results showed that after one week of continuous administration, the actual IOP in the high IOP rabbit treated with carteolol hydrochloride group, the netarsudil dimesylate group, and FC-CN was reduced to approximately 29.93%, 31.53%, and 42.65%, respectively. These results indicated that the combination of carteolol and netarsudil dimesylate increased the IOP-lowering effect. The IOP change in FC-CN-TKN group was 1.48 times higher than that in FC-CN, demonstrating that efficacy improvement correlates with HPMC-mediated viscosity enhancement, which prolonged the precorneal residence time and amplified transcorneal drug flux, and ultimately improved the IOP reduction effect.

### 3.7. Protective effect on RGCs of FC-CN-TKN

Glaucoma is a neurodegenerative disease that eventually leads to blindness due to the permanent death of RGCs and the loss of optic nerve fibers (Almasieh et al. 2012). Thus, the protection of RGCs from death is vital importance for glaucoma patients. Extensive studies have reported that ROCK inhibitors can increase ocular blood flow, thus slowing the progression of glaucomatous optic neuropathy by directly increasing the perfusion of the retina and optic disk (Tanna and Johnson 2018; Ou-Yang et al. 2020). Building upon these mechanistic insights, we quantified RGC survival through histomorphometric analysis (H&E staining) and apoptotic profiling (TUNEL assay) in a validated glaucoma model (Figures S4, S5, 6C). The apoptosis rates of RGCs in the control group, model group, carteolol hydrochloride group, netarsudil dimesylate group, FC-CN group, and FC-CN-TKN group were 2.93%, 17.41%, 6.73%, 5.08%, 4.98%, and 3.3%, respectively. This finding indicated that the combination of carteolol and netarsudil dimesylate could provide better protection for the optic nerve than monotherapy. Importantly, the number of surviving RGCs in FC-CN-TKN was more than that in FC-CN group, indicating that the addition of a thickener can prolong the ocular retention time, improve the drug penetration into the cornea, and ultimately gain a better optic nerve protection.

## 4. Conclusion

Current glaucoma therapies continue to face significant challenges in clinical practice, including frequent dosing requirements, preservative-related ocular toxicity, and limited therapeutic efficacy—factors that collectively compromise patient adherence and long-term outcomes. The once-daily FC-CN-TKN presented in this study has the ability to treat glaucoma by lowering the IOP with great efficacy and improving patient compliance. FC-CN-TKN uses a methanesulfonic acid-driven simple and easy one-pot pH-adjusting solubilization strategy that overcomes drug‒drug incompatibility and solubility limitations. Importantly, the addition of the thickener HPMC prolonged the ocular retention time of FC-CN-TKN, increased the IOP-lowering effect and protected the optic nerves. In conclusion, FC-CN-TKN, with good stability and safety, has a great potential in controlling IOP and protecting the optic nerve from damage, thus representing an alternative to the current treatments of glaucoma.

## Supplementary Material

Response to reviewers.docx Response to reviewers.docx

Supplementary Materials.docx

Supplementary material Author Checklist.pdf

## Data Availability

The data supporting this study's findings are available from the corresponding author upon reasonable request.
