# Supplementary material for: Preparation of a new fixed-combination carteolol-netarsudil ophthalmic solution via one-pot pH-adjusting solubilization strategy for glaucoma treatment
Source: Drug Deliv. 2026 Aug 15;33(1):2708376. doi: 10.1080/10717544.2026.2708376 (PMC13479515; doi:10.1080/10717544.2026.2708376)
Supplement: Supplementary file 2 [file IDRD_A_2708376_SM8422.docx]

Manuscript No.: UDRD-2025-0297 (Submission No.: 258038855)

Title: Preparation of a new fixed-combination carteolol-netarsudil ophthalmic solution via one-pot pH-adjusting solubilization strategy for glaucoma treatment

Dear Editors,

Thank you very much for your comment dated on 3-Dec-2025.

We would like to express our sincere gratitude to the reviewers for their insightful feedback and valuable suggestions, which have greatly contributed to improving our manuscript. In response, we have diligently revised the manuscript. All modifications have been incorporated into the revised version and are highlighted for your convenience. Our point-by-point responses to the comments are provided below.

We are looking forward to your final decision.

With kind regards,

Yours sincerely,

Prof. Qikun Jiang

Wuya College of Innovation

Shenyang Pharmaceutical University

**Response to Reviewers’ Comments**

**We have highlighted our updated text in red color and please kindly help to review.**

**Reviewer: 1**

**Comments to the Author**

**The author successfully addressed the incompatibility between commercially available drugs (carteolol and netarsudil) by employing one-pot pH-adjusting solubilization strategy, resulting in the preparation of a stable compound ophthalmic solution. This work is interesting and quite novel, laying a solid foundation for the development of new therapeutics for glaucoma in clinical practice. There are some comments that may further improve the quality of this manuscript.**

**1. It is noted that the introduction underscores the adverse effects of prostaglandin drugs, which presumably led to their exclusion from the fixed combination. Could the authors elaborate on the specific reasons for their drug selection, including why other classes, for instance, cholinomimetic agents, were not pursued?**

**Response: Thank you for your questions. Although cholinomimetic drugs were beneficial in certain forms of glaucoma, its adverse effect profile, especially in phakic individuals, often limited its use [1]. In the clinical studies, significant reductions in aqueous humor production were seen with carteolol in patients with primary open-angle glaucoma (POAG) and it did not alter corneal sensitivity, tear secretion or pupil diameter. Unlike timolol or betaxolol, carteolol also appeared to improve perfusion of the optic nerve head [2]. Besides, once daily dosing of netarsudil demonstrated significant IOP lowering and a favorable safety profile in subjects with glaucoma and ocular hypertension in clinical studies [3]. More importantly, it had a protective effect on the optic nerve [4]. The combination of carteolol and netarsudil dimesylate exerted a synergistic action to lower intraocular pressure, characterized by well-defined targets, pronounced efficacy, optic nerve protection and a favorable safety profile. These attributes led to their selection as the active ingredients in our fixed-combination ophthalmic solution.**

**2. The authors used methanesulfonic acid as a solubilizer. Could the author please elaborate on the rationale for selecting this specific agent over other solubilizing agents**

**Response: Thank you for your questions. Methanesulfonic acid was a common pharmaceutical excipient, used to improve the solubility, stability, and bioavailability of drugs [5–7]. Many commercially available drugs were provided in the form of mesylate salts, such as Betahistine mesylate, Pefloxacin mesylate, and netarsudil mesylate, etc. The use of commercial netarsudil mesylate ophthalmic solution (Rhopressa^®^ and Rocklatan^®^) proved the rationality and safety of methanesulfonic acid use in the eyes. Therefore, we chose methanesulfonic acid to solubilize the free base of carteolol by adjusting pH.**

**3. The authors should include the measurement of a commercially available product like Mikelan® as a reference standard to confirm the reliability of the osmolarity instrument.**

**Response: Thank you for your questions. We have supplemented this part of the experiment and included the results in the revised manuscript. The results showed that the instrument could accurately detect the 295 mOsm/kg osmolality of Mikelan^®^. Also, the osmolality of FC-CN-TKN showed no significance when the content of mannitol was 1.2 w/v% compared with Mikelan^®^ (page 20, lines 15-17 and Fig. S3). These conclusions were consistent with previous ones.**

**4. The labeling of the horizontal axis in figure 4 appears inconsistent and should be verified.**

**Response: Thank you for your questions. We carefully examined Fig. 4 and corrected the errors in the revised manuscript.**

**5. A dosage of 40 μL per eye was administered to the rabbits in the pharmacokinetic and pharmacodynamic studies. Please explain why this dosage was applied.**

**Response: Thank you for your questions.** **In most of the literatures, ophthalmic solution was administered around 30-50 μL per human or rabbit eye [8–11]. Meanwhile, we found when 35-40 μL** **ophthalmic solution were placed in the rabbit's lower conjunctival sac,** **ophthalmic solution rarely** **escaped from the can thus causing** **waste. In order to avoid the difference caused by the different amount of** **canthus loss and maximize the amount of ophthalmic solution exposure to the eye, we chose 40 μL per eye to conduct vivo experiments.**

**6. The authors state that viscosity enhancers can reduce dosing frequency by prolonging retention time. However, the final formulation still requires daily dosing in pharmacodynamic experiment, which appears contradictory. Please explain this discrepancy.**

**Response: Thank you for your questions. The recommended administration regimen for commercial carteolol hydrochloride ophthalmic solution (Mikelan^®^) was one drop, twice daily (Figure 1). By incorporating viscosity-enhancing agents, formulation FC-CN-TKN demonstrated superior therapeutic efficacy with only a single daily dose, offering a potential reduction in dosing frequency compared to the standard therapy.**

**Figure 1. The usage instructions of Mikelan^®^ ophthalmic solution**

**7. It would be helpful to clarify the intended clinical indication for the fixed-combination ophthalmic solution. Specifically, is it targeted for open-angle glaucoma or angle-closure glaucoma, or perhaps both?**

**Response: Thank you for your questions. The core difference between open-angle glaucoma and angle-closure glaucoma lied in whether the angle structure was obstructed. The angle structure of open-angle glaucoma was open, but the discharge of aqueous humor was obstructed. The angle stenosis or closure of angle-closure glaucoma resulted in physical obstruction of the aqueous humor drainage channel [12,13]. Carteolol hydrochloride was a non-selective β-adrenergic blocker, exerts potent IOP-lowering effects through suppression of aqueous humor (AH) production. Most of its research focused on open-angle glaucoma [14]. Netarsudil was able to increase the outflow of AH to exert the IOP-lowering effect via Rho kinase inhibition. Therefore, its therapeutic effect on angle-closure glaucoma was limited. Its usage instructions clearly indicated that it was suitable for open angle glaucoma (Figure 2). FC-CN-TKN featured a combined mechanism of action. It integrated two complementary pharmacological effects to lower intraocular pressure: reducing aqueous humor production while concurrently increasing its outflow, thus possibly having better therapeutic effects on open-angle glaucoma.**

**Figure 2. The usage instructions of Rhopressa^®^ ophthalmic solution**

**We have highlighted our updated text in red color and please kindly help to review.**

**Reviewer: 2**

**Comments to the Author**

**1. In section 2.3, reword this as "We found that carteolol hydrochloride causes netarsudil dimethanesulfonate to precipitate at approximately pH 5.0 for better clarity" also add Therefore, carteolol (the free base) was used in this study to..."**

**Response: Thank you for your questions. We replaced these sentences in the revised manuscript (section 2.3, page 11, lines 1-4).**

**2. 40-70% is inconsistent. Make sure wherever this is mentioned, correct and write properly.**

**Response: Thank you for your questions. We carefully examined the manuscript and corrected the errors in the revised manuscript (page 14, line 2 and line 9).**

**3. In 2.7.2, Please confirm the urethane concentration and dosing units (mg/kg or g/kg?).**

**Response: Thank you for your questions. We carefully examined this section and found the dosage unit was written incorrectly. The proper dose of urethane was 0.8 g/kg. We corrected it in the revised manuscript (section 2.7.2, page 15, lines 14-16).**

**4. What is the purpose of left-eye untreated controls if the disease is only induced in the right eye? Why not use a dedicated normal control group instead?**

**Response: Thank you for your questions. In animal experiments,** **using the right eye as the experimental group and the left eye as the control group was a common design [15,16]. The core advantage of this design was the use of animals themselves as controls, which could effectively control individual differences such as genetic background, age, gender, and environmental factors, making the results more accurate and reliable. In addition, this design could reduce the required number of animals and comply with the 3R principle (Replacement, Reduction and Refinement) [17].**

**5. Please add w/v% wherever necessary and correct.**

**Response: Thank you for your questions. We carefully examined the manuscript and added w/v% wherever necessary in the revised manuscript (page 11, lines 14 and 18; page 15, lines 15 and 18; page 19, lines 1, 11, 12 and 16; page 20, lines 15, 19 and 20; page 21, lines 1 and 3; Fig. 3D).**

**6. In section 3.6, how many sections and regions per eye were analyzed for RGC counts? and what criteria were used to identify RGCs in H&E?**

**Response: Thank you for your questions. For the first question, when analyzing the number of RGCs, 3 sections and 5 regions were analyzed for each eye. For the second question, the axons of retinal ganglion cells (RGCs) formed the innermost retinal layer, known as the retinal nerve fibre layer. Approximately one million of these nerve fibres converged to form the human optic nerve, exiting the eye at the optic disc (or optic nerve head). Across studied species so far, the inner plexiform layer (IPL), where RGCs established intraretinal connections, was subdivided into structurally and functionally distinct sublaminae (Figure 3). In this layered organization, ON RGCs extended their dendritic arbors exclusively within the inner sublamina (sublamina b) of the IPL, whereas OFF RGCs stratified in the outer sublamina (sublamina a) [18,19].**

**Figure 3. Schematic representation of the retina. GCL, ganglion cell layer; IPL, inner plexiform layer; INL, inner nuclear layer; OPL, outer plexiform layer; ONL, outer nuclear layer; RGC, retinal ganglion cell; A, amacrine cell; B, bipolar cell; H, horizontal cell; P, photoreceptor. RGCs can be functionally divided into ON and OFF subclasses. The dendritic arbors of ON and OFF RGCs stratify in different sublaminae (a, OFF or b, ON) of the IPL [19].**

**Taking Fig. S4A in the supplementary materials for an example, the area indicated by the red arrow corresponded to the ganglion cell layer (Figure 4), so the retinal ganglion cells could be quantitatively analyzed.**

**Figure 3. Schematic diagram of the ganglion cell layer in Fig. 4A (red arrow).**

**7. Please mention and show statistical analysis in figures 3 A and B and mention it in figure captions too.**

**Response: Thank you for your questions. The matter was resolved by re-analyzing the data sets using a one-way ANOVA test. The results in Fig. 3A showed that the use of thickeners carbomer, CMC-Na, and sodium alginate significantly reduced the content of netarsudil dimesylate in the solution. The results in Fig. 3B showed that when the HPMC model was SH-E50, there was no significant difference in dynamic viscosity compared to commercially available artificial tears (Aierming^®^ and Zhendi^®^). Therefore, HPMC SH-E50 was chosen as thickener for further research. These conclusions were consistent with previous ones.**

**References**

**Reviewer: 1**

**1. Dikopf MS, Vajaranant TS, Edward DP. Topical treatment of glaucoma: established and emerging pharmacology. Expert Opin Pharmacother. 2017;18:885–98. https://doi.org/10.1080/14656566.2017.1328498**

**2. Henness S, Swainston Harrison T, Keating GM. Ocular Carteolol. Drugs Aging. 2007;24:509–28. https://doi.org/10.2165/00002512-200724060-00007**

**3. Lin C-W, Sherman B, Moore LA, Laethem CL, Lu D-W, Pattabiraman PP, et al. Discovery and Preclinical Development of Netarsudil, a Novel Ocular Hypotensive Agent for the Treatment of Glaucoma. Journal of Ocular Pharmacology and Therapeutics. 2018;34:40–51. https://doi.org/10.1089/jop.2017.0023**

**4. Kitaoka Y, Sase K, Tsukahara C, Fujita N, Arizono I, Kogo J, et al. Axonal Protection by Netarsudil, a ROCK Inhibitor, Is Linked to an AMPK-Autophagy Pathway in TNF-Induced Optic Nerve Degeneration. Investigative Opthalmology & Visual Science. 2022;63:4. https://doi.org/10.1167/iovs.63.1.4**

**5. Nema S, Washkuhn RJ, Brendel RJ. Excipients and their use in injectable products. PDA J Pharm Sci Technol. 1997;51:166–71.**

**6. Bae SK, Park JB, Seo JH, Choi W-K, Park S, Sung YJ, et al. Improved oral absorption of cilostazol via sulfonate salt formation with mesylate and&amp;nbsp;besylate. Drug Des Devel Ther. 2015;3961. https://doi.org/10.2147/DDDT.S87687**

**7. Serajuddin ATM. Salt formation to improve drug solubility. Adv Drug Deliv Rev. 2007;59:603–16. https://doi.org/10.1016/j.addr.2007.05.010**

**8. Calissendorff B, Sjöquist B, Högberg G, Grunge-Lowerud A. Bioavailability in the Human Eye of a Fixed Combination of Latanoprost and Timolol Compared to Monotherapy. Journal of Ocular Pharmacology and Therapeutics. 2002;18:127–31. https://doi.org/10.1089/108076802317373888**

**9. Halder A, Khopade AJ. Pharmacokinetics and Pharmacology of Latanoprost 0.005% without Benzalkonium Chloride Vs Latanoprost 0.005% with Benzalkonium Chloride in New Zealand White Rabbits and Beagles. Curr Eye Res. 2021;46:1031–7. https://doi.org/10.1080/02713683.2020.1855660**

**10. Cantor LB, Hoop J, Wudunn D, Yung C-W, Catoira Y, Valluri S, et al. Levels of bimatoprost acid in the aqueous humour after bimatoprost treatment of patients with cataract. British Journal of Ophthalmology. 2007;91:629–32. https://doi.org/10.1136/bjo.2006.110155**

**11. Zammataro A, Civiale C, Saletti R, Foti S. Development and validation of a liquid chromatography/electrospray ionization tandem mass spectrometry method for the quantification of latanoprost free acid in rabbit aqueous humor and ciliary body. Journal of Mass Spectrometry. 2011;46:1168–74. https://doi.org/10.1002/jms.2004**

**12. Weinreb RN, Leung CKS, Crowston JG, Medeiros FA, Friedman DS, Wiggs JL, et al. Primary open-angle glaucoma. Nat Rev Dis Primers. 2016;2:16067. https://doi.org/10.1038/nrdp.2016.67**

**13. Wang Y, Guo Y, Zhang Y, Huang S, Zhong Y. Differences and Similarities Between Primary Open Angle Glaucoma and Primary Angle-Closure Glaucoma. Eye Brain. 2024;Volume 16:39–54. https://doi.org/10.2147/EB.S472920**

**14. Patton GN, Lee HJ. Chemical Insights into Topical Agents in Intraocular Pressure Management: From Glaucoma Etiopathology to Therapeutic Approaches. Pharmaceutics. 2024;16:274. https://doi.org/10.3390/pharmaceutics16020274**

**Reviewer: 2**

**15. Panchal SS, Mehta AA, Santani DD. Effect of potassium channel openers in acute and chronic models of glaucoma. Taiwan J Ophthalmol. 2016;6:131–5. https://doi.org/10.1016/j.tjo.2016.05.006**

**16. Veronica E Giordano, Sergio E Hernandez-Da Mota, Tania N Adabache-Guel, Armando Castillejos-Chevez, Sonia Corredor-Casas, Samantha M Salinas-Longoria, et al. Safety of intravitreal quinupristin/dalfopristin in an animal model. Int J Ophthalmol. 2016; https://doi.org/10.18240/ijo.2016.03.08**

**17. Díaz L, Zambrano E, Flores ME, Contreras M, Crispín JC, Alemán G, et al. Ethical Considerations in Animal Research: The Principle of 3R’s. Rev Invest Clin. 2020;73:199–209. https://doi.org/10.24875/RIC.20000380**

**18. Weinreb RN, Khaw PT. Primary open-angle glaucoma. The Lancet. 2004;363:1711–20. https://doi.org/10.1016/S0140-6736(04)16257-0**

**19. Sernagor E, Eglen SJ, Wong ROL. Development of Retinal Ganglion Cell Structure and Function. Prog Retin Eye Res. 2001;20:139–74. https://doi.org/10.1016/S1350-9462(00)00024-0**
