# Supplementary material for: Preparation of a new fixed-combination carteolol-netarsudil ophthalmic solution via one-pot pH-adjusting solubilization strategy for glaucoma treatment
Source: Drug Deliv. 2026 Aug 15;33(1):2708376. doi: 10.1080/10717544.2026.2708376 (PMC13479515; doi:10.1080/10717544.2026.2708376)
Supplement: Supplementary file 3 [file IDRD_A_2708376_SM8423.docx]

**Supplementary Materials**

**Preparation of a new fixed-combination carteolol-netarsudil ophthalmic solution via one-pot pH-adjusting solubilization strategy** **for glaucoma treatment**

**Authors:**

Dian Jiao ^a,^ ^1^, Tao Wang ^a,^ ^1^, Tu Lu ^a, b, 1^, Xiaohua Ran ^a,^ ^1^, Yanxin Li ^a^, Yanhua Liu ^c^, Chunyang Zhao ^d^, Tianhong Zhang ^a^, Xiaoli Ma ^b, *^, He-Nan Liu ^e, *^, Jin Sun ^a, *^, Qikun Jiang ^a, f*^

**Affiliation:**

^a^ Department of Pharmaceutics, Wuya College of Innovation, Shenyang Pharmaceutical University, Shenyang, China.

^b^ Department of Ophthalmology, The First Hospital of China Medical University, Shenyang, China

^c^ Department of Pharmaceutics, School of Pharmacy, Ningxia Medical University, Yinchuan, China.

^d^ Department of Ophthalmology, Shengjing Hospital of China Medical University, Shenyang, China.

^e^ Department of Pharmacy, The First Hospital of China Medical University, 155 Nanjing North Street, Shenyang, China.

^f^ Joint International Research Laboratory of Intelligent Drug Delivery Systems, Ministry of Education, Shenyang, China.

**^*^Correspondence:**

Prof. Qikun Jiang, Prof. Jin Sun, Prof. Xiaoli Ma, and Prof. He-Nan Liu

Wuya College of Innovation, Shenyang Pharmaceutical University, 103 Wenhua Road, Shenyang 110016, China

E-mail address: jiangqk_student@aliyun.com (Q. Jiang)

sunjin@syphu.edu.cn (J. Sun)

liuhn@cmu.edu.cn (H. Liu)

20022083@cmu.edu.cn (X. Ma)

**Supplementary Tables**

**Table S1** The dynamic viscosity of commercial artificial tears and different types of HPMC (0.5%) in fixed-combination carteolol-netarsudil ophthalmic solution. (n = 3)

| Item | Dynamic Viscosity (mPa·s) |
| --- | --- |
| HPMC ophthalmic solution  (Tradename: Aierming^®^) | 7.651 ± 0.151 |
| HPMC ophthalmic solution  (Tradename: Zhendi^®^) | 7.612 ± 0.126 |
| HPMC 60RT15 in FC-CN | 6.731 ± 0.373 |
| HPMC SH-E3 in FC-CN | 5.830 ± 0.219 |
| HPMC SH-E5 in FC-CN | 6.005 ± 0.193 |
| HPMC SH-E50 in FC-CN | 7.522 ± 0.104 |
| HPMC SH-K4M in FC-CN | 30.22 ± 0.086 |
| HPMC SH-K100M in FC-CN | 146.2 ± 1.452 |

**Table S2** The prescribing information of commercial netarsudil dimesylate ophthalmic solution (Tradename: Rhopressa^®^) and carteolol hydrochloride ophthalmic solution (Tradename: Mikelan^®^).

| Prescription | Netarsudil dimesylate ophthalmic solution | Carteolol hydrochloride ophthalmic solution |
| --- | --- | --- |
| Name | Netarsudil ophthalmic solution | Carteolol hydrochloride ophthalmic solution |
| Appearance | Transparent liquid | Transparent liquid |
| Active pharmaceutical ingredients | Netarsudil mesylate  (0.0285%) | Carteolol hydrochloride  (2%) |
| Buffers | Boric acid  (0.05%) | Phosphate buffer  (-) |
| Tonicity agents | Mannitol  (4.7%) | Sodium chloride  (-) |
| pH adjusting agents | Sodium hydroxide | Sodium hydroxide |
| Preservatives | Benzalkonium chloride  (0.015%) | Benzalkonium chloride  (0.02%) |
| pH | 5.0 | 6.2-7.2 |
| Osmolality | 295 mOsm/kg | 295 mOsm/kg |
| Drug specifications | 2.5 mL:0.5 mg | 2.5 mL:50 mg |
| Storage | 2-8℃ | 2-8℃ |

**Table S3** The AH pharmacokinetic parameters of carteolol in rabbits after single ophthalmic administration (40 μL per eye). (Mean ± SD, n = 3)

|  | Carteolol hydrochloride | FC-CN | FC-CN-TKN |
| --- | --- | --- | --- |
| C_max_  (ng/mL) | 639.4 ± 74.0 | 646.3 ± 76.8 | 507.9 ± 56.2 |
| T_max_  (ng/mL) | 1.0 ± 0.0 | 1.0 ± 0.0 | 1.5 ± 0.0 |
| AUC_(0-t)_  (ng·h/mL) | 1556 ± 164 | 1573 ± 171 | 2873 ± 435 |
| t_1/2_  (h) | 1.46 ± 0.06 | 1.46 ± 0.13 | 3.51 ± 1.23 |

**Table S4** The AH pharmacokinetic parameters of netarsudil-M1 in rabbits after single ophthalmic administration (40 μL per eye). (Mean ± SD, n = 3)

|  | Netarsudil dimesylate | FC-CN | FC-CN-TKN |
| --- | --- | --- | --- |
| C_max_  (ng/mL) | 484.1 ± 151.5 | 516.7 ± 160.6 | 350.3 ± 77.8 |
| T_max_  (ng/mL) | 0.5 ± 0.0 | 0.5 ± 0.0 | 1.5 ± 0.0 |
| AUC_(0-t)_  (ng·h/mL) | 930 ± 248.1 | 946 ± 253.4 | 1865 ± 339.4 |
| t_1/2_  (h) | 1.50 ± 0.14 | 1.56 ± 0.27 | 6.46 ± 2.21 |

**Table S5** Pharmacodynamic parameters after topical application of carteolol hydrochloride, netarsudil dimesylate, FC-CN and FC-CN-TKN for 7 days in Japanese white rabbits with the compound carbomer solution-induced ocular hypertension (mean ± SD, n = 3). **p < 0.01, ***p < 0.001, ****p < 0.0001 compared with model group, #p < 0.05, ##p < 0.01, ###p < 0.001 compared with FC-CN-TKN group.

| Group | IOP Day 0  (mmHg) | Actual IOP Day 7  (mmHg) | IOP Changes  (mmHg) |
| --- | --- | --- | --- |
| Control | 6.25 ± 0.96 | 7.75 ± 1.26 | 1.50 ± 1.91 |
| Model | 28.25 ± 2.99 | 33.75 ± 5.73 | 5.50 ± 4.51 |
| Carteolol hydrochloride | 32.50 ± 4.51 | 22.50 ± 3.42^** ###^ | -10.00 ± 5.60^*** ##^ |
| Netarsudil dimesylate | 26.50 ± 3.54 | 19.50 ± 0.71^** ##^ | -12.25 ± 4.24^* ###^ |
| FC-CN | 33.33 ± 3.21 | 19.00 ± 3.61^*** ##^ | -14.33 ± 2.08^**** #^ |
| FC-CN-TKN | 33.75 ± 6.34 | 10.5 ± 3.32^****^ | -23.25 ± 5.73^****^ |

**Supplementary Figures**

**Fig. S1** The appearance of carteolol in water without methanesulfonic acid (A), and with methanesulfonic acid (B) at pH 5.0 aqueous solution

**Fig. S2** The appearance of FC-CN containing different thickeners: carbomer (A), HPMC (B), CMC-Na (C), HA (D), Sodium Alginate (E) and PVP (F).

**Fig. S3** The measured osmolality of FC-CN-TKN showed no significance compared with commercial Mikelan^®^ (295 mOsm/kg) when containing 1.2 w/v% mannitol (n = 3, n.s.: no significance).

**Fig. S4** H&E staining of retinal sections in control group (A), model group (B), carteolol hydrochloride group (C), netarsudil dimesylate group (D), FC-CN group (E) and FC-CN-TKN group (F).

**Fig. S5** TUNEL detection of retina in control group (A), model group (B), carteolol hydrochloride group (C), netarsudil dimesylate group (D), FC-CN group (E) and FC-CN-TKN group (F).
